# Artificial intelligence in surgery: the emergency surgeon’s perspective (the ARIES project)

**DOI:** 10.1007/s44250-022-00014-6

**Published:** 2022-12-06

**Authors:** Belinda De Simone, Elie Chouillard, Andrew A. Gumbs, Tyler J. Loftus, Haytham Kaafarani, Fausto Catena

**Affiliations:** 1 Department of Emergency, Digestive and Metabolic Minimally Invasive Surgery, Poissy and St Germain en Laye Hospitals, Poissy, France; 2 Department of Surgery, University of Florida Health, Gainesville, USA; 3 Division of Trauma, Emergency Surgery and Surgical Critical Care, Massachusetts General Hospital, Boston, USA; 4 Department of Emergency and General Surgery, Level I Trauma Center, Bufalini Hospital, Cesena, Italy

**Keywords:** Artificial Intelligence, Machine learning, Decision-making, ARIES project, Emergency surgery, Autonomous action, Robotic surgery, Digital vision

## Abstract

Artificial Intelligence (AI) has been developed and implemented in healthcare with the valuable potential to reduce health, social, and economic inequities, help actualize universal health coverage, and improve health outcomes on a global scale. The application of AI in emergency surgery settings could improve clinical practice and operating rooms management by promoting consistent, high-quality decision making while preserving the importance of bedside assessment and human intuition as well as respect for human rights and equitable surgical care, but ethical and legal issues are slowing down surgeons’ enthusiasm. Emergency surgeons are aware that prioritizing education, increasing the availability of high AI technologies for emergency and trauma surgery, and funding to support research projects that use AI to provide decision support in the operating room are crucial to create an emergency “intelligent” surgery.

## Background

Artificial Intelligence (AI) has been developed and implemented worldwide in many fields.

In healthcare, AI has the valuable potential to reduce health, social, and economic inequities, help actualize universal health coverage, and improve health outcomes on a global scale.

The COVID-19 pandemic was characterized in its early period by a tremendous number of patients needing hospital admission, leading to collapsing healthcare systems globally. Healthcare system strain was exacerbated by limited hospital resources and the low availability of COVID tests and personal protective equipment (PPE). These limitations increased the interest of governments, private companies, and public healthcare systems in developing AI systems to improve the management of patients.

In this time of need, AI tools and new digital technologies allowed institutions, medical staff, and stakeholders to clinically manage a large number of patients and large volume healthcare data, both in real-time and remotely [1].

AI tools implemented on big data from electronic health records used heterogeneous, massive, and complex datasets that require integration of different types of information identifying clusters and correlations, and extracting value to transform large volumes data into information and knowledge in the form of predictive models to improve decision-making quality [2].

This became technically possible by building big data platforms to store and integrate high-capacity and high-diversity biological, clinical, environmental, and lifestyle information related to health status collected from individuals and population at one or more time points for real-time demand [3].

Advances in AI have helped big data technology to progress beyond the simple analysis of the traditional hypothesis and query paradigm.

It is well accepted that traditional predictive analytics and clinical decision-support systems in surgery have a compromised clinical utility because of the time-consuming nature of manual data management and the suboptimal accuracy that is inherent in traditional clinical decision-support systems that conform to rules rather than learning from data. On the contrary, AI refers to computer systems that mimic human cognitive functions such as learning and problem-solving, which can be performed with or without human supervision [4].

Machine learning (ML) is a subfield of AI that enables machines to learn and make predictions by recognizing patterns to support rational human decision-making and it is increasingly being applied to the medical fields. ML allows a computer to utilize human labelling of the data (supervised learning) or the structure detected in the data itself (unsupervised learning) to explain or make predictions about the data with fewer explicit human instructions to do so.

Deep Learning (DL) is a type of ML in which computer systems learn and represent highly dimensional data by adjusting weighted associations among input variables via a layered hierarchy modelled on the anatomic structure of neurons and the neural cortex. DL networks are neural networks comprised of many layers and may learn more complex and subtle patterns than simple one or two-layer neural networks. An algorithm optimizes and updates weights as the model is trained to achieve the strongest possible association between input and output layers [4].

## Artificial intelligence for surgery: enthusiasm or disillusions?

AI has been progressively implemented in clinical practice in supporting physicians, and recently surgeons, in improving their clinical decision-making, diagnostics, and surgical procedures, in predicting and limiting human errors in critical highly stressful situations through preoperative risk stratification, intra-operative monitoring of surgical procedures, and postoperative phases of care.

For example, in oncology, research has demonstrated that ML applications can be of great help for the diagnosis or detection of cancer [5]. In the intensive care unit, with the implementation of the "internet of things" (IoT), which includes electronic systems used for communication between individuals with the final goal of enabling a variety of things/objects present in the environment to be connected and to interact and cooperate anytime, anyplace, with anything and anyone, the monitoring of the patient becomes “real-time” even remotely [6–8].

AI in surgery is developing more slowly than in medicine and radiology, which may be partially attributable to the high stakes and complexity of surgical decision-making, dominated by hypothetical-deductive reasoning, based on the unpredictable interaction with the patient, human intuition, and the environment.

AI research in surgery involves algorithms and the development of semi-autonomously acting devices and robotics capable of doing interventional gestures/actions in different surgical procedures by a different interaction with the surgeon, but always under his/her supervision.

This interaction and supervision had been classified into 6 levels, as summarized in Table 1 [9].

**Table 1:** Classification of autonomy in Artificial Intelligence surgery

Level of autonomy of AI tools	Operator’s interaction
0 = no autonomy	The surgeon performs all tasks including monitoring, generating performance options, selecting the option to perform and executing the decision made
1 = Robot assistance	The surgeon keeps continuous control of the system and robot provides certain assistance
2 = Task autonomy	The surgeon keeps discrete control of the system and the robot can perform certain operator-initiated tasks automatically
3 = Conditional autonomy	The surgeon selects and approves a surgical plan, and the robot performs procedure automatically but with close surgical oversight by human
4 = High autonomy	Robot ia able to make decisions but under the supervision of a qualified operator
5 = Full automation	No human needs to be in the loop, and the robot can perform an entire surgical procedure

Simple energy devices and power staplers used routinely in surgical practice may be considered as “intelligent” devices, in fact, they use algorithms to calculate hundreds of thousands of data points per second to determine the optimal coagulation velocity without movement or gesture of the surgeon, or they are provided of sensors that block the stapling action if the tissue is too thick or not thick enough, without interaction with the surgeon.

Because of all these technological innovations, the enthusiasm towards AI developments is growing among surgeons.

**Fig. 1:**
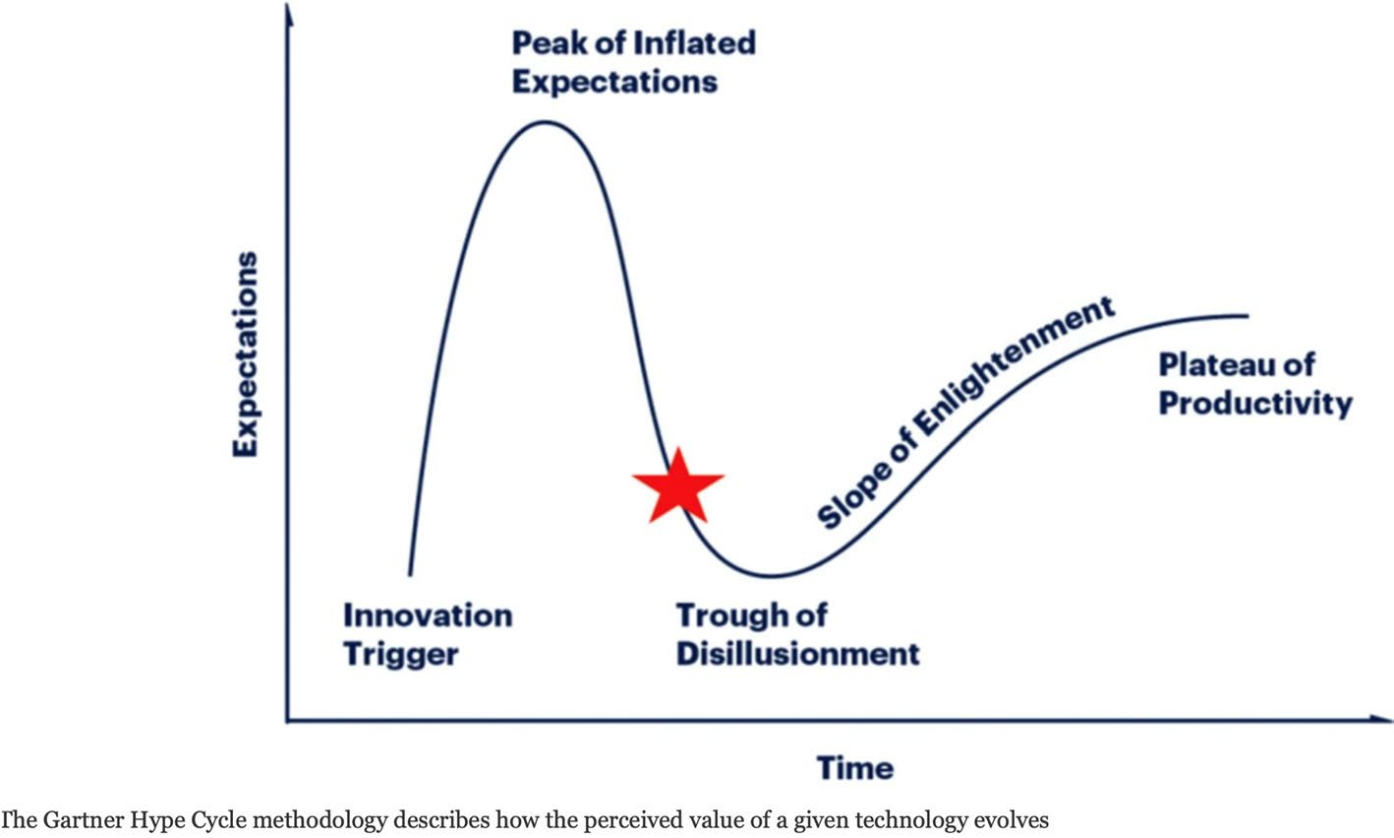
The Gartner Hype Cycle for Artificial Intelligence tools in surgery

Despite this high surgeons’ motivation, AI research in surgery is following a classic S-shaped curve, going through the “Peak of Inflated Expectations” to the “Trough of Disillusionment” according to Rogers’ *Diffusion of Innovations* theory [10] (Fig. 1).

## Overview on artificial intelligence tools for surgery

At present, the support of AI in surgical practice is mainly based on ML and computer vision (CV), which enables computers and systems to derive meaningful information from digital images, videos and other visual inputs and to take actions or make recommendations based on that information, augmented reality, and anatomical segmentation.

CV softwares can improve surgeons' performance in critical situations such as difficult cholecystectomies [11, 12] using computational methods and focuses on sub-components of the human visual system such as object detection, identification, motion extraction, or spatial understanding and have 3 main applications: the surgical process understanding, computer-aided detection, and computer-assisted navigation [13].

For example, surgical robots such as the da Vinci Surgical System allow surgeons to perform surgery from a remote booth with telemanipulation to control the arms of the robot and to use 3-D stereo endoscopes.

AI segmentation and localization contribute to safe tool-tissue interaction and are essential to visually guided manipulation tasks, significantly improving surgical gesture flexibility, accuracy, and reliability over hand-crafted tool tracking methods [13, 14].

Moreover, fusing multiple pre-operative and intra-operative imaging modalities in an augmented reality (AR) view of the surgical site enhances surgical navigation by vision-based AR systems that display the fused information to the surgeon [15].

Panesar et al. discussed also the role of the internet or mobile platforms, controlled by AI, in providing surgical expertise remotely, allowing surgical robots to perform surgeries, alone but under the supervision of a connected surgeon, or guiding surgeons in difficult surgical procedures, where appropriate resources are unavailable or access is lacking, such as in war or during a disaster [16].

Despite these extraordinary results reported in literature, significant problems persist in the implementation of these vision-based AR systems, such as adding contextual information to the visualization (e.g., identifying anatomical structures and critical surgical areas and detecting surgical phases and complications) and ensuring robust localization [13].

Several pilot studies' results are promising, but AI algorithms and digital vision in the OR are not yet ready for large-scale implementation in clinical practice. Despite promising results from the pre-clinical development causal inference models, AI is not yet able to detect causal relationships in data at a necessary level for clinical practice.

AI systems inside the operating room (OR) may have a promising future in monitoring OR access, occupation, and practice in evolving technology like the OR black box, a multiport synchronized data capture and analytic platform [17, 18].

The OR Black Box technology, which leverages DL algorithms, continuously and simultaneously recorded intraoperative audiovisual data and physiological parameters from both patients and health care professionals, and multiple other sensors and devices [17]. In-OR video is captured using a wide-angle cameras, and intracorporeal video is collected from the laparoscope or robotic camera or light-mounted or wearable cameras in open surgical procedures [17].

All data are stored on a secure server for further analysis based on procedural steps, disruptive environmental and organizational factors, OR team technical and nontechnical skills, surgeon physiological stress, intraoperative errors, events, and rectification processes [17].

The main aim is to develop a “surgical control tower” that, using AI, can monitor, analyse and support workflows and outcomes in real-time, improving operating room management, surgical quality and safety, decreasing healthcare costs [18].

Inside and outside of OR, decision-making is the most difficult and high responsibility surgeons’tasks. Surgeons have to evaluate the patient and all clinical informations correlated, in a short time and often in a stressful setting, to take complex decisions.This mental processus is dominated by hypothetical deductive reasoning and individual judgment and depends on personal behaviour, the patient and the environment, with high variability and risk of errors.

Loftus et al. [19] showed that traditional clinical-decision support systems are time-consuming because of manual data management and do not consider the nonlinear relationship among multiple non-static variables, decreasing accuracy.

AI models, fed with live streaming intraoperative and electronic health record data (EHR), integrated with bedside assessment and human intuition could improve critical decision-making.

For example, according to ML methods, the Predictive OpTimal Trees in Emergency Surgery Risk (POTTER) [20] smartphone application was developed such as novel, interactive, and nonlinear risk calculator for predicting postoperative mortality, morbidity, and other specific surgical complications, to assist in real-time emergency surgeons in preoperative evaluation of the patient.

This calculator showed higher accuracy compared to American Society of Anesthesiologists (ASA) classification, Emergency Surgery Score (ESS), and ACS Surgical Risk Calculator [20]. On the same AI technology, the Trauma Outcome Predictor (TOP) smartphone application was developed to predict mortality and morbidity in trauma patients and support emergency surgeon in balancing risks and benefits with patients and relatives [21].

Main ethical issue with the implementation of these AI tools in clinical practice is concerning the validity and generalisability of ML prediction that depends on the accuracy, comprehensiveness and representativity of the data integrated.

## The Artificial Intelligence in emergency and trauma surgery project: the emergency surgeons’ perspective

Before COVID pandemic broke out, emergency surgeons were already aware of the crucial role of implementing technology in clinical practice to improve the management of patients presenting acute abdominal pain when they proposed the Video-Consulting Emergency (VCE) protocol as a simple tool to improve the decision-making process between an on-site emergency physician and a remote acute care surgeon using a smartphone, the FaceTime application, and the Acute Abdominal Decision Making (AADM®) model [22, 23].

The Artificial intelligence in Emergency and trauma Surgery (ARIES) project was conceived to increase research and availability of AI systems for emergency and trauma surgery [24].

The first step of this project was to carry out an international web survey endorsed by the World Society of Emergency Surgery to assess the knowledge, attitude and practices of AI in emergency and acute care surgery. It was shown that there is a deep interest among emergency surgeons in investigating AI implementation in the emergency setting, in supporting decision-making, and in developing autonomous actions in surgery, to improve clinical and surgical outcomes.

**Fig. 2:**
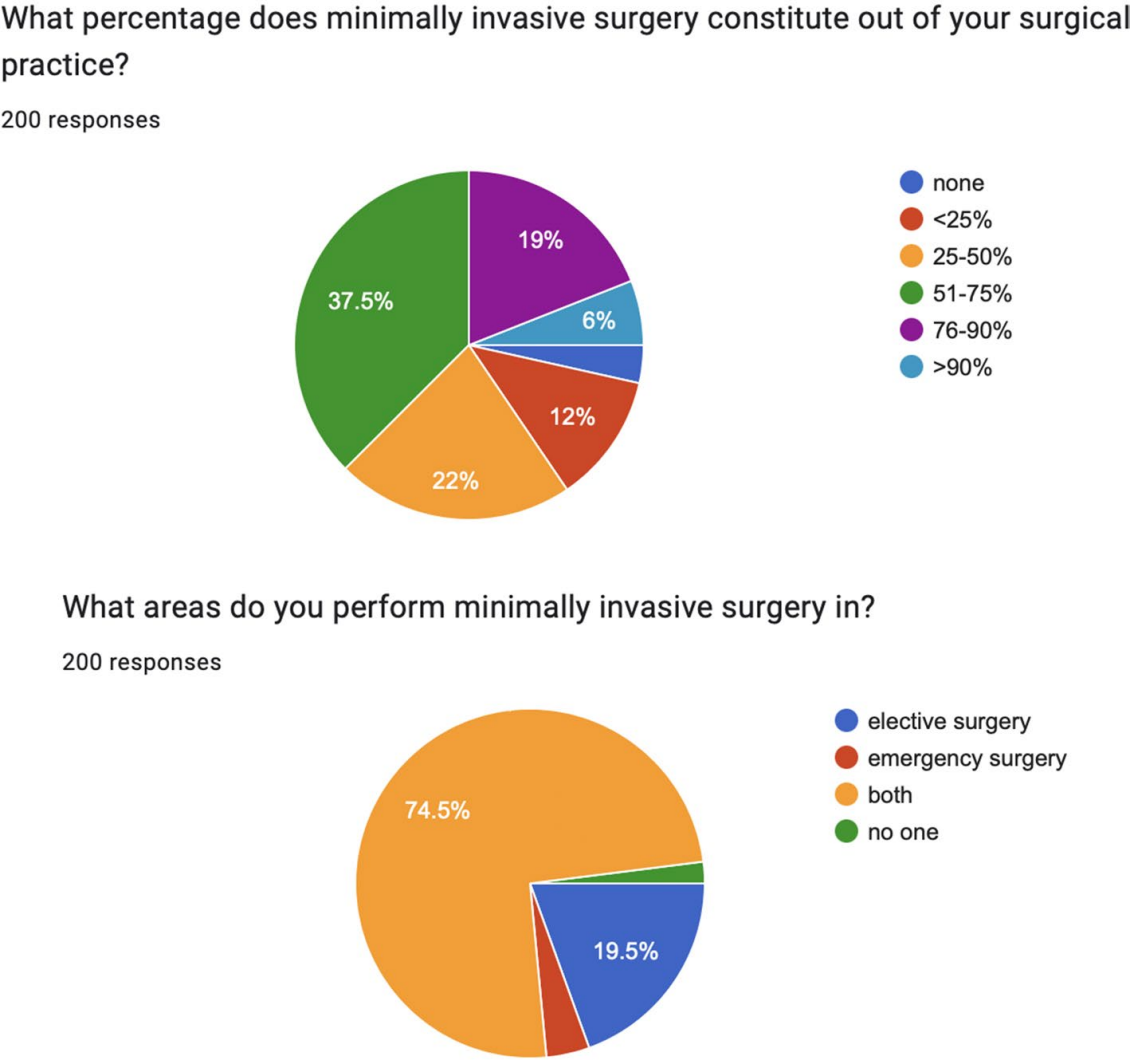
Minimally Invasive Surgery and Emergency Surgery, from the Artificial Intelligence in Emergency and trauma Surgery (ARIES) survey results

**Fig. 3:**
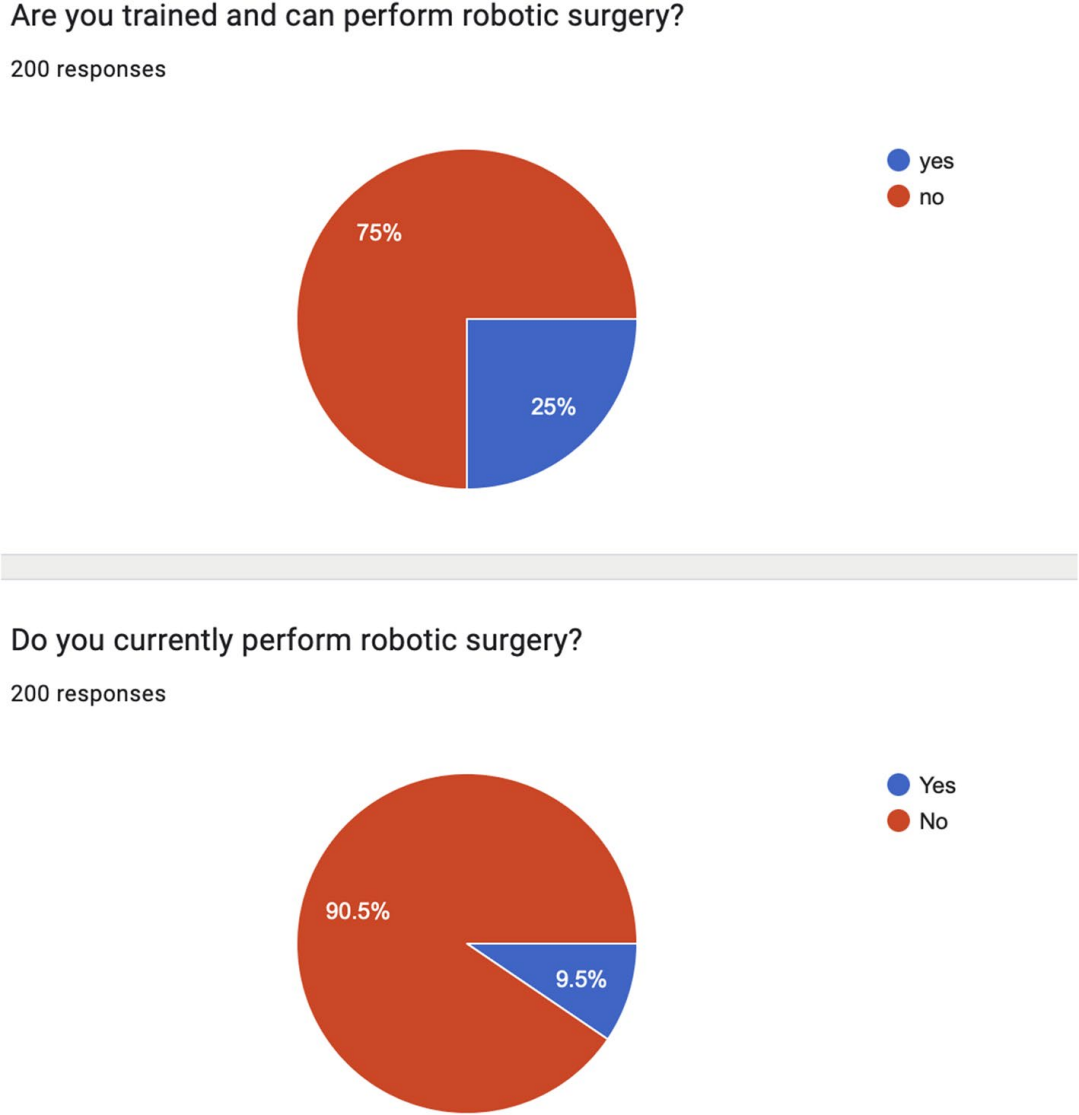
Robotic surgery and emergency surgery, from Artificial Intelligence in Emergency and trauma Surgery (ARIES) survey results

From this survey, significant data emerged (Figs. 2, 3), such as:

- For only 37% (75/200) of responders, minimally invasive surgery (MIS) accounts for more than half of their surgical activity;
- MIS is performed for both elective and emergency procedures for 74% (149/200) of responders;
- 90% (181/200) of emergency surgeons do not perform robotic surgery;
- 75% (150/200) of emergency surgeons are not trained in robotic surgery;
- 63% (127/200) of emergency surgeons do not have robotic surgery platforms in their institution;
- 55% (110/200) of emergency surgeons have 3D vision experience;
- 54% (109/200) of emergency surgeons use 3D vision tools only for elective surgical procedures.

So, despite the advancements in AI developing systems, there is still a problem of availability and accessibility of high technology tools that integrate with AI for emergency and trauma surgery in highly stressful settings where monitoring decision-making and surgeons’ performance has the potential to mitigate harm from preventable errors.

## Artificial intelligence: main issues and emergency surgeons’ contribution

Moreover, the adoption of innovative technology always involves early adopters (i.e., people who represent opinion leaders, enjoy leadership roles, and embrace change opportunities) and late adopters (i.e., people skeptical of change and will only adopt an innovation after it has been tried by the majority and proven successful). During the innovation process, when an individual is motivated to reduce uncertainty about the advantages and disadvantages of an innovation, it is important to emphasize the ethical and legal challenges.

Litvin et al. [25] assessed the effectiveness of artificial neural network (ANN) based decision support systems in emergency surgery and reported that these tools are affected by confounders that are inherent to the complexity and heterogeneity of emergency surgical diseases and patients. The effectiveness of ANN under these conditions depends on:

- The quality and reliability of medical information;
- The lack of transparency in the decision-making process by the intellectual core of the system, according to the “black box” principle that refers to ML models that give an answer or make a decision without explaining or showing how they did it;
- The selection and development of personnel capable of effectively using and maintaining intelligent systems;
- The high cost of projects;
- The security of data storage.

O’Sullivan et al. [26] reviewed the literature about AI and autonomous robotic surgery, focusing on legal responsibility, and classified responsibility into accountability, liability, and culpability, emphasizing that supervision of AI by human surgeons and physicians remains mandatory.

Legal issues concerning AI tools also include privacy, cybercrime and ethics to ensure that new technologies are used responsibly, including human representatives, to build trust between humans and AI techniques.

To address these concerns, many initiatives and principles documents were provided, including the Partnership on AI ([27] https://gpai.ai/), OpenAI ([28] https://openai.com/about/), the Foundation for Responsible Robotics ([29] https://responsiblerobotics.org/), the Ethics and Governance of AI Initiative ([30] https://aiethicsinitiative.org/), the Montréal Declaration for Responsible Development of AI [31], and the Principles for Accountable Algorithms ([32] https://www.fatml.org/resources/principles-for-accountable-algorithms).

Furthermore, there are other barriers to the widespread implementation of AI in health care such as unawareness of the topic or solutions, lack of implementation knowledge by the medical professionals and their workplace supporters, concerns about ethics or privacy, and insufficient technical and funding support.

Emergency and trauma surgeons are aware of the importance of recording, collecting high-quality surgical and clinical data (health data, images, videos) and of disposing of large, international, secured databases (registers) to train algorithms with high reliability and accuracy.

Emergency and trauma surgeons strongly believe that the implementation of AI in emergency and trauma settings is a major opportunity to improve outcomes, and are ready to be involved in future research projects about AI application in supporting decision-making and surgical procedures.

The ARIES survey [19] showed that emergency surgeons’ ability to adopt new technologies is significantly correlated with interest and expectations, consequently, increasing interest and grounding the implementation process in surgeons’ expectations with usability testing and user-centered design has the potential to overcome disillusionment and barriers.

We believe that advancements in AI for surgery require close collaborative working groups (algorithm developers, engineers, physicians, surgeons, stakeholders, politics) and the development of non-surgical skills aiming to obtain an optimal clinical application of AI in surgery, with women and men from diverse training and sociodemographic backgrounds contributing equally to permit the deepest understanding of AI in surgery in a manner that represents the diversity and uniqueness of the patients we serve.

According to this, emergency and trauma surgeons responded that prioritizing education, increasing the availability of high AI technologies for emergency and trauma surgery, and funding to support research projects that use AI to provide decision support in the OR in difficult and high stressful situations are crucial to develop an emergency “intelligent” surgery.

Emergency and trauma surgeons have led the charge when it comes to creating and disseminating simulation-based education, most notably with the ATLS (Advanced Trauma Life Support) course. The creation of even more advanced AI-enabled simulations could enable even more significant reductions in preventable errors and delays in diagnosis and therapeutic administration, resulting in improved outcomes during trauma resuscitations.

## Conclusions

Education, accessibility to high technology devices and AI tools, and research funding are the keys to create an emergency “intelligent” surgery and improve the management of patients in difficult and stressful settings.

The application of AI in emergency and trauma surgery settings could improve clinical practice by promoting consistent, high-quality decision making while preserving the importance of bedside assessment and human intuition as well as respect for human rights and equitable surgical care.

Emergency surgeons are ready to contribute with international, high quality, clinical and surgical data (registers) to develop inclusive and effective algorithms and to work on an intelligence emergency and trauma surgery.

## Data Availability

Not applicable.

## Abbreviations

ARIES: Artificial Intelligence in emergency surgery and trauma surgery
ML: Machine learning
DL: Deep learning
OR: Operating room
CV: Computer vision
EHR: Electronic health record data
